# Natural history of preexisting AAV5 antibodies in adults with hemophilia B during the lead-in of the etranacogene dezaparvovec phase 3 study

**DOI:** 10.1016/j.omtm.2025.101568

**Published:** 2025-08-18

**Authors:** Robert Klamroth, Michael Recht, Nigel S. Key, Wolfgang Miesbach, Steven W. Pipe, Radoslaw Kaczmarek, Douglass Drelich, Blanca Salazar, Sandra Le Quellec, Paul E. Monahan, Nicholas Galante, Paul van der Valk, Jacqueline Tarrant

**Affiliations:** 1 Vivantes Klinikum, Landsberger Allee 49, 10249 Berlin, Germany; 2 Institute of Experimental Hematology and Transfusion Medicine, University Hospital Bonn, Medical Faculty, University of Bonn, Sigmund-Freud-Strasse 25, 53127 Bonn, Germany; 3 Yale School of Medicine, 333 Cedar Street, New Haven, CT 06510, USA; 4 National Bleeding Disorders Foundation, 1230 Avenue of the Americas 16th Floor, New York, NY 10020, USA; 5 UNC Blood Research Center and Department of Medicine, University of North Carolina, 8008 Mary Ellen Jones Building, CB7035 Chapel Hill, NC 27514, USA; 6 Department of Haemostaseology and Haemophilia, University Hospital Frankfurt, Theodor-Stern-Kai 7, 60596 Frankfurt, Germany; 7 Departments of Pediatrics and Pathology, University of Michigan, 1500 East Medical Drive, Ann Arbor, MI 48109-5718, USA; 8 Department of Pediatrics, Indiana University School of Medicine, 1044 West Walnut Street, R4-116, Indianapolis, IN 46202, USA; 9 CSL Behring, 1020 First Avenue, King of Prussia, PA 61501, USA; 10 CSL Behring Europe, Philipp-Reis-Strasse 2, 65795 Hattersheim am Main, Germany; 11 Center for Benign Haematology, Thrombosis and Haemostasis, University Medical Center Utrecht, University Utrecht, Van Creveldkliniek, Heidelberglaan 100, 3584 CX Utrecht, the Netherlands

**Keywords:** adeno-associated virus serotype 5, hemophilia B, etranacogene dezaparvovec, neutralizing antibody, anti-AAV5, factor IX

## Abstract

Testing for binding or neutralizing antibodies (NAbs) to adeno-associated virus (AAV) is part of the laboratory assessment of people with hemophilia considering AAV-based gene therapy. We evaluated the natural history of NAb titers to AAV serotype 5 (AAV5) in adult males ≥18 years old with hemophilia B (factor IX ≤ 2%) during the lead-in period of a phase 3 trial prior to the infusion of etranacogene dezaparvovec to characterize NAb in addition to immunoglobulin G (IgG) and immunoglobulin M (IgM) anti-AAV5 binding antibody changes over time. At screening, 48% (32/67) of enrolled participants had detectable NAbs (NAb+) with a median titer of 58 (range: 9–3,440). Participant-specific lead-in periods differed and included discontinuers (median duration: 240 days; range: 1–360). The median intra-participant coefficient of variation of NAb titer over time was 25% (range: 2%–154%). NAb seropositivity was associated with older age (*p* = 0.0065). For participants with detectable anti-AAV5 NAbs and IgG, there was a high correlation of titers at each visit (median r = 0.96; range: 0.92–0.99). IgM anti-AAV5 antibodies were detectable in only 9% of participants, and seroconversion was infrequent. In conclusion, AAV5 NAb test results were consistent over 6 months, which informs the timing of NAb screening when considering gene therapy for hemophilia B.

## Graphical abstract

**Figure:**
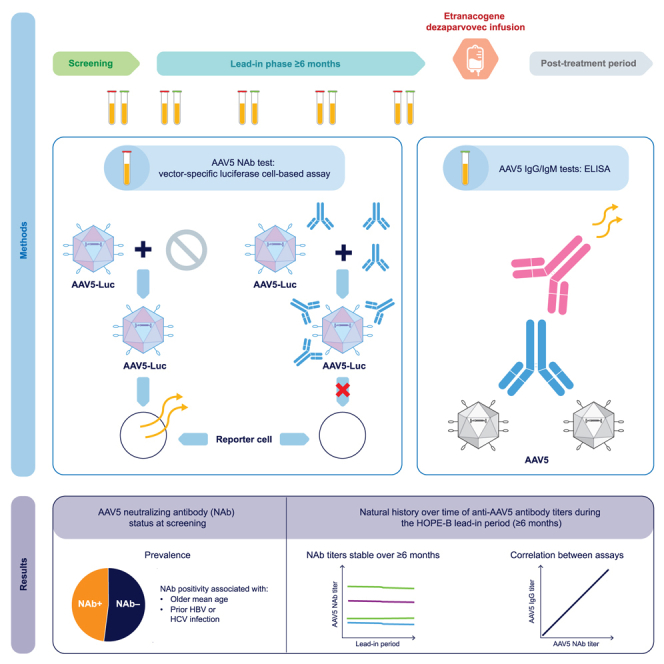

## Teaser

Adeno-associated virus serotype 5 neutralizing and IgG antibody levels were correlated and remained stable over 6 months prior to dosing in the HOPE-B trial of etranacogene dezaparvovec for hemophilia B, providing valuable guidance on timing for antibody screening when considering gene therapy.

## Introduction

Hemophilia B is an X-linked bleeding disorder caused by inheritable mutations in the *F9* gene that result in varying levels of deficiency of clotting factor IX activity. Gene therapy is one approach used to treat this genetic deficiency. Recombinant adeno-associated virus (AAV) gene delivery systems have been successfully deployed in gene therapies that are marketed or in development to treat hemophilia B. Etranacogene dezaparvovec is an AAV serotype 5 (AAV5)-based gene therapy with a codon-optimized gene expression cassette encoding the naturally occurring human factor IX Padua (R338L) variant.^1^^,^^2^^,^^3^

AAV is a non-replicative single-stranded DNA parvovirus with 13 human or nonhuman primate serotypes grouped into 6 clades (A–F) and two phylogenetically distinct isolates comprising AAV5 and another group (AAV serotypes 4, 11, and 12), based on similarities in sequence and antigenicity.^4^ AAV5 is divergent from the common clades, with only 51%–59% amino acid sequence homology of capsid viral proteins 1 and 3 (compared with ∼85% homology between AAV serotype 2, AAV serotype 8, and AAV serotype rh10) and structural differences in capsid variable regions involved in transduction and antigenicity.^4^^,^^5^^,^^6^ Several serotypes have been used in gene therapy for hemophilia, including AAV2 (clade B), 5 (distinct clone), 6 (clade A), 8 (clade E), a variant of rh74 (clade E), and engineered variants of naturally occurring serotypes.^7^^,^^8^

As wild-type AAV occurs naturally in the environment, people can become exposed and develop antibodies to the viral capsid as part of the immune response to the virus.^9^^,^^10^^,^^11^ The similarity between wild-type and recombinant AAVs of the same serotype and among different serotypes can result in pre-existing cross-reacting antibodies, potentially inhibiting transduction of the target tissue by the viral vector.^12^^,^^13^ Consequently, for many AAV serotypes, people with pre-existing anti-AAV antibodies may be excluded from participating in most gene therapy clinical trials, potentially limiting access to commercially available treatments as well. However, in the etranacogene dezaparvovec trials that also enrolled people with hemophilia B who were AAV5 neutralizing antibody (NAb)-positive (NAb+), effective *in vivo* gene transduction was demonstrated.^1^^,^^14^ Additionally, in the pivotal study of valoctocogene roxaparvovec, an AAV5-based factor VIII gene therapy for hemophilia A, three people who were negative at screening became positive for AAV5 total binding antibodies (titers <20–91) immediately prior to dosing, and 11 people positive for AAV5 neutralization in the AAV5 transduction inhibition (TI) assay (titers of 1–188, note that this may include some people with NAbs and uncharacterized non-antibody factors) were successfully transduced.^15^

Antibodies binding to the AAV capsid are commonly measured using enzyme-linked immunosorbent assay (ELISA) methodology.^16^ However, not all antibodies binding an AAV vector have the ability to inhibit transduction of the target cells.^9^ To assess this characteristic, a TI assay was deployed in the development of etranacogene dezaparvovec. This cell-based, *in vitro* assay quantifies the extent to which serum from a person with hemophilia B inhibits the transduction of a cell line by an AAV5 serotype vector containing a gene cassette encoding a reporter protein for detection. In both ELISA and cell-based assays, the titer is semi-quantitative and is the inverse of the serum dilution, whereby the higher the antibody titer, the higher the serum dilution. Although providing an assessment of the relative neutralizing capacity of serum from a person with hemophilia B, TI assays are non-standardized, so titers should not be directly compared across laboratories.^16^^,^^17^^,^^18^

As people living with hemophilia B with pre-existing AAV5 NAbs can otherwise be eligible for AAV5 gene therapy, we sought to characterize the humoral immune profile and biological variability of anti-AAV5 antibodies in adults with hemophilia B using data obtained in the observational screening and lead-in periods of the phase 3 HOPE-B pivotal clinical trial (NCT03569691) over at least 6 months.^1^

## Results

### Increased prevalence of AAV5 NAb seropositivity is associated with older age

A total of 67 adult males with hemophilia B were enrolled into the lead-in period (Table 1). Participants were predominantly White, and approximately one-third (38.8%) lived in the United States, while two-thirds lived in Western Europe. The seroprevalence of NAb+ participants at screening was 47.8%, and the median NAb+ titer was 58 (range: 9–3,440). NAb positivity was associated with an older mean age (*p* = 0.0065), and participants were likely to have had prior hepatitis B or C virus infections (*p* = 0.0013). When adjusted for age, the association with prior hepatitis B or C virus infections and NAb serostatus remained significant (*p* = 0.04), although age did explain some of the relationship. HIV+ status was also more frequent in participants who were NAb+, although very few HIV+ participants were enrolled overall (*n* = 4; 6.0%).

**Table 1:** Demographic characteristics in the lead-in safety population (*N* = 67)

	NAb+^a^	NAb–	Overall study population (*N* = 67)
NAb status, *n* (%)	32 (47.8)	35 (52.2)	–
Age, mean (SD), years	48 (17.5)	38 (13.2)	43 (16.2)
Range	19–78	21–73	19–78
Race, *n* (%)			
White	21 (65.6)	29 (82.9)	50 (74.6)
Other	3 (9.4)	4 (11.4)	7 (10.4)
Asian	3 (9.4)	0	3 (4.5)
Black/African American	2 (6.3)	0	2 (3.0)
Missing	3 (9.4)	2 (5.7)	5 (7.5)
Country, *n* (%)			
USA	15 (46.9)	11 (31.4)	26 (38.8)
Netherlands	9 (28.1)	7 (20.0)	16 (23.9)
Other^b^	3 (9.4)	9 (25.7)	12 (17.9)
UK	3 (9.4)	4 (11.4)	7 (10.4)
Belgium	2 (6.3)	4 (11.4)	6 (9.0)
BMI, mean (SD), kg/m^2,^^c^	27.9 (6.3)	27.5 (4.5)	27.7 (5.4)
Severity of hemophilia B at the time of diagnosis, *n* (%)			
Severe (FIX plasma level <1 IU/dL)	26 (81.3)	30 (85.7)	56 (83.6)
Moderately severe (FIX plasma level (1–2 IU/dL)	6 (18.8)	5 (14.3)	11 (16.4)
HIV+, *n* (%)	3 (9.4)	1 (2.9)	4 (6.0)
Hepatitis B			
Prior or ongoing^d^^,^^e^	10 (31.3)	3 (8.6)	13 (19.4)
Prior (resolved)	10 (100.0)	3 (100.0)	13 (19.4)
Positive at screening assessment	0	0	0
Hepatitis C			
Prior or ongoing^d^^,^^e^	24 (75.0)	14 (40.0)	38 (56.7)
Prior (resolved)	23 (95.8)	14 (100.0)	37 (97.4)
Positive at screening assessment^d^^,^^f^	1 (4.2)	0	1 (2.6)

BMI, body mass index; FIX, factor IX; HCV, hepatitic C virus; NAb, neutralizing antibody.

a At screening.

b Other countries included Italy, Germany, Ireland, and Sweden.

c *n* = 66.

d Denominator for percentages is the number of participants in the prior or ongoing subgroup.

e Prior or ongoing per reported medical history.

f Participants positive at screening had detectable HCV RNA. This participant was positive at screening while undergoing HCV anti-viral therapy with glecaprevir/pibrentasvir and was negative at the L-Final visit (−21 days before etranacogene dezaparvovec administration).

**Figure 1:**
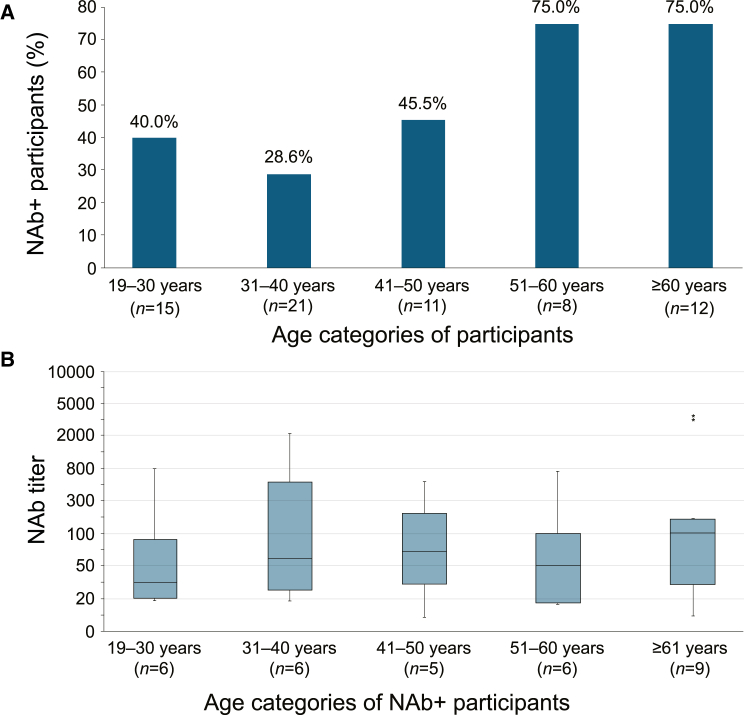
NAb seroprevalence and titer per age category NAb+ status by age category in the overall population (A; *N* = 67) and NAb titer by age category of NAb+ participants (B; *n* = 32) ∗NAb titer measure considered to be an outlier. NAb, neutralizing antibody; NAb+, neutralizing antibody-positive.

The relationship of NAb status and NAb titer with age was further explored in age groups of 10-year intervals (Figures 1A and 1B). Seropositivity was notably more frequent in participants aged 51 years and older. However, there was no association between increasing age and titer.

### AAV5 NAb titers are consistent over multiple months

**Figure 2:**
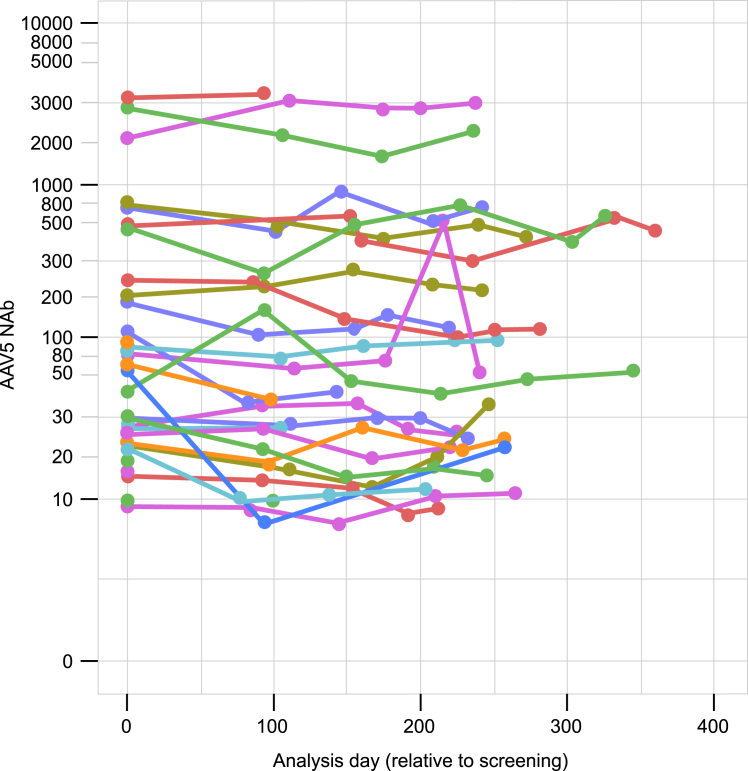
Plot of NAb values for each participant who was NAb+ at screening and during the lead-in period of at least 6 months (*n* = 26) Please note that 6 participants were only NAb+ at screening and did not subsequently have positive NAb values during the rest of the lead-in period. AAV5, adeno-associated virus serotype 5; NAb, neutralizing antibody; NAb+, neutralizing antibody-positive.

Participant NAb data were collected during the lead-in period, the duration of which differed for each participant and included discontinuers (median lead-in duration period: 240 days; range: 1–360 days). Despite a wide range of NAb titers among participants, titers remained stable for each patient and without large fluctuations over time (Figure 2). The median intra-participant coefficient of variation (CV) of NAb titers over time was moderate (CV: 25%; range: 2%–154%). For the 19 participants who were NAb+ and remained on study for the complete lead-in period of at least 6 months (median duration of 246 days; range: 216–360 days), there was high consistency between the screening titer and the titer immediately prior to receiving gene therapy (Table 2). This cohort did not include 2 participants who seroconverted during the lead-in period and who are described later in the text.

**Table 2:** AAV5 NAb titer agreement between screening and pre-dose baseline visit in the lead-in safety population (*n* = 19)

	Screening	Pre-dose baseline visit^b^
*n*^a^	19	19
Mean (SD)	292.0 (482.25)	325.5 (730.21)
Median (min, max)	88.2 (8.8, 2,020.4)	57.8 (8.5, 3,212.3)
ICC (95% CI)^c^	0.89 (0.741, 0.957)	

AAV5, adeno-associated virus serotype 5; ICC, intraclass correlation coefficient; *n*, number of participants (paired); NAb, neutralizing antibody.

a Only patients with paired data were included.

b The pre-dose baseline visit was the baseline visit during which etranacogene dezaparvovec was administered.

c The ICC was calculated using a type 3 sums of squares from an analysis of variance model.

Similarly, NAb status showed high agreement over this period (kappa [95% confidence interval (CI)]: 0.7 [0.51–0.89]). Six participants (6/67, 9%), all with NAb titers <25 at screening, seroreverted from NAb+ to NAb− by the end of the lead-in period (data not shown). Four participants (6.0%) had an isolated change in NAb status during the lead-in period. Several of these participants had low NAb titers (7, 8, 20, 67) that were close to the NAb assay limit of detection (LoD).

### AAV5 NAbs are highly correlated with AAV5 IgG binding antibodies

**Figure 3:**
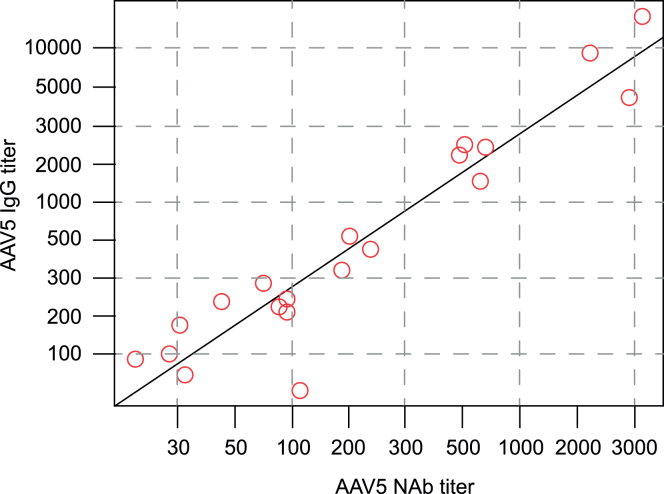
Correlation of NAb and IgG titers at screening in the lead-in safety population (*n* = 20) ^a^Please note that two patients did not have quantifiable levels of IgG and were therefore omitted from this figure. AAV5, adeno-associated virus serotype 5; IgG, immunoglobulin G; NAb, neutralizing antibody.

At multiple time points during screening and lead-in, neutralizing and immunoglobulin G (IgG) or immunoglobulin M (IgM) binding antibodies to AAV5 were measured using specific assays for each analyte. Detectable AAV5 NAbs and IgG binding antibodies were highly correlated at each time point during the lead-in period (median r = 0.96; range: 0.92–0.99). The relationship at screening is presented in Figure 3 (r = 0.95; 95% CI: 0.871, 0.980). The concordance of the assays was influenced by differing assay sensitivity; the NAb assay was optimized for sensitivity and validated and run in a CAP-CLIA (The College of American Pathologists-Clinical Laboratory Improvement Amendments)-certified laboratory, whereas the total IgG ELISA was a validated research use-only assay. At screening, all participants who were IgG+ were also NAb+; discordance was attributable to the 10 (14.9%) participants who were NAb+ but IgG−. The NAb titers detected in all of the NAb+/IgG− patients were relatively low, and all were lower than the median NAb+ titer of 58. No participant was both NAb− and IgG+, and around half of the participants (*n* = 35; 52.2%) were both NAb− and IgG−. At the screening assessment, two participants had positive IgG levels in the IgG anti-AAV5 confirmatory assay but with a titer below LoD in the quantitative titering assay. Both participants’ NAb titer values at screening were also relatively low (titer <20).

### AAV5 IgM binding antibodies and seroconversion were infrequent

Few participants had detectable anti-AAV5 IgM (6/67, 9%), all of whom were NAb+ and only 3 had detectable anti-AAV5 IgG. IgM was generally transient and at low titer (within one dilution step of the analytical LoD), and in only one case was IgM detected throughout the entire screening and lead-in period. Participants who were IgM+ ranged in age from 34 to 69 years old, distributed among multiple age groups.

**Figure 4:**
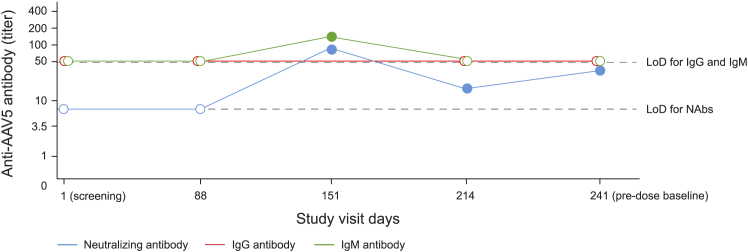
Longitudinal NAb, IgM, and IgG measurements at visits during the lead-in phase for the participant who experienced seroconversion Please note that circles indicate a titer value <LoD; dots indicate a titer value ≥ LoD. The final lead-in visit occurred within 28 days of etranacogene dezaparvovec administration (pre-dose baseline). AAV5, adeno-associated virus serotype 5; LoD, limit of detection; IgG, immunoglobulin G; IgM, immunoglobulin M; NAb, neutralizing antibody.

One participant seroconverted from NAb− to NAb+ 4 months after screening, consistent with a new infection and accompanied by a contemporaneous transient spike in IgM (Figure 4). IgG was undetectable during this period. Another participant may have seroconverted, shifting from NAb− at screening to NAb+ 8 months later. However, the NAb titer (13.7) was close to the LoD, and anti-AAV5 IgM or IgG was not detected at any time point.

## Discussion

Gene therapy is an innovative approach that holds great promise for treating many different conditions. However, understanding the impact of NAbs on gene transduction is key to harnessing the clinical promise of this technology. For several therapies, even low-level NAb positivity renders people ineligible for participation in clinical trials. For those therapies for which NAb positivity is not a contraindication, extremely high titers likely impact transduction efficiency, with a resultant impact on clinical efficacy.^1^ Screening for NAbs is therefore a key component of evaluating an individual’s eligibility and suitability for commercial gene therapy. To date, very little has been known about the natural history of NAb titers over time, leading to uncertainty regarding the optimal timing for NAb screening when considering gene therapy. This can lead to multiple testing and inconsistent timing of screening prior to planned treatment. Therefore, understanding not only the epidemiology of NAbs but also their kinetics over time is key to ensuring that only suitable people with hemophilia B receive gene therapy.

As the HOPE-B clinical trial allowed enrollment regardless of pre-existing AAV5 NAb titers,^1^ this represents a unique opportunity for detailed characterization of the natural history of antibodies over a median of 8 months in adult men. The 48% seroprevalence of AAV5 NAb+ in this population is comparable to the range of NAb seropositivity (25%–53%^19^^,^^20^^,^^21^) or binding anti-AAV5 (22%–36%^10^^,^^21^) previously documented in people with hemophilia.

In our study, the prevalence of NAb seropositivity was associated with older age, particularly in participants who were 50 years and older; this compares to a similar association in people with hemophilia B who were approximately 40 years and older in other studies.^10^^,^^19^^,^^21^ However, age and titer were not significantly associated. The increased frequency of NAb positivity in patients with a history of hepatitis B or C has been reported previously.^21^ Additionally, we found that the association with hepatitis B or C was influenced by older age, although the NAb serostatus associated with viral hepatitis B or C remained when controlled for age. The relationship between AAV5 serostatus and viral hepatitis is not clear; viral transmission because of exposure to contaminated blood products prior to improved safety measures instituted in the 1990s may in part contribute.^22^ Additionally, parvovirus B19 exposure in plasma-derived products has been documented more recently, and as AAV is a member of the Parvoviridae family, this therefore provides another possibility for AAV exposure.^23^ We did not collect sufficient medical history data reporting plasma product use to explore this potential relationship with NAb status.

Comprehensive longitudinal analysis of anti-AAV5 NAbs over several months demonstrated that NAb titers and/or serostatus were stable over time, as observed previously in limited studies of anti-AAV NAbs over months to years.^10^^,^^13^^,^^20^^,^^24^ The few participants who fluctuated between being NAb+ and NAb− over several time points had titers generally close to the analytical LoD, consistent with technical variability in NAb AAV assays.^13^^,^^20^^,^^24^ Approximately 10% of participants seroreverted from NAb positivity to NAb negativity. Although we did not measure cross-reactivity to other AAV serotypes in this study, low and transient NAb titers could represent cross-reactive NAbs.^11^ People can be seropositive for multiple AAV serotypes, whether due to coinfection or subsequent infections of multiple AAV serotypes or because antibodies may be cross-reactive to multiple AAV serotypes; the relative concentration and neutralizing ability of these antibodies may differ between AAV serotypes.^9^^,^^19^^,^^25^ For example, it has been reported that neutralizing titers of cross-reactive AAV antibodies were lower than that of the suspected primary AAV serotype,^13^^,^^26^ and donors with low-titer NAbs to an AAV serotype were less likely to neutralize other serotypes.^9^

Aspects of assay design (explored further), such as the limit of quantification (where an assay is precise and linear), serum dilution scheme, and specificity, can also influence cross-reactivity observations.^27^ Alternatively, relatively lower immunogenicity of the AAV5 serotype compared to some other serotypes (e.g., AAV2) may explain low and transient NAb titers. Seroprevalence surveys from multiple groups have repeatedly demonstrated that anti-AAV5 NAbs are on the lower end of the range for prevalence and/or have lower titers compared to other AAV serotypes deployed in gene therapy.^9^^,^^10^^,^^18^^,^^20^^,^^27^^,^^28^^,^^29^^,^^30^^,^^31^^,^^32^ Seroconversion from NAb− to NAb+ occurred in <5% of participants, a similar incidence to previous reports.^13^^,^^20^^,^^24^ IgM was generally undetectable using our multi-tiered bioanalytical approach, which included a separate confirmatory assay to reduce false positives.^33^ All participants with IgM confirmed as positive had low titers (<150, i.e., within one serum dilution step) and were NAb+. In most cases, antibodies to AAV were class switched from IgM to IgG.^13^ We note that the IgM AAV5 observations are constrained by assay sensitivity, and we cannot rule out a higher incidence of IgM positivity if a more sensitive assay were deployed. In practice, these data support the suggestion that, a titer level, whether low or high, will be steady over several months, with infrequent seroconversion from NAb− to NAb+, and there is limited value in measuring the IgM antibody subclass.

A strong correlation of NAb titer with IgG anti-AAV5 titer over the measuring range of both assays across multiple time points was observed. This correlation analysis was constrained by the lack of sensitivity of the total IgG ELISA.

A high correlation has previously been observed for AAV5 serological assays,^9^^,^^34^ although the correlation was not always significant.^21^ Pan et al. demonstrated that indirect binding assays for AAV6 had a higher correlation to NAb than the bridging ELISA format,^18^ which may explain the discrepancy in our high correlations for AAV5 compared to another report.^21^ When this is examined as concordance of NAb serostatus compared to IgG (or total Ig), anti-AAV serostatus is highly influenced by the sensitivity of the assays and in some instances by the ELISA format.^18^ In this study, we observed 85% concordance between NAb and IgG serostatus (i.e., NAb+IgG+ or NAb−IgG−) to AAV5, which is within the range others have noted for AAV5 (72%–86%) or AAV6 (92%). In our study, discordance, i.e., NAb+IgG− exclusively, was entirely consistent with the higher relative analytical sensitivity of the NAb assay compared to the IgG anti-AAV5 assay.

Antibodies to AAV can have several functional attributes, including binding to the capsid, which potentially permits redirection and uptake by the macrophage-monocyte system, complement binding, and neutralization activities by inhibiting attachment and/or uptake of the capsid by receptors on the cell and endosomal trafficking.^17^ Non-antibody neutralizing factors, such as potentially soluble AAV receptors, proteins, or interfering drugs, generally have only low titers in the TI assay.^17^^,^^34^^,^^35^^,^^36^ Assay parameters and methods can have a profound influence on the quantification of NAbs, which can result in different rates of seropositivity and titers.^18^^,^^29^^,^^37^ This can include the cell type and permissiveness to transduction, AAV serotype, reporter vector expression system, luminescence reader, concentration and ratio of cells to vector (i.e., multiplicity of infection [MOI]), empty capsid content of the reporter vector, and serum volume, in addition to each protocol step, which are often manual and take place over 2–3 days.^17^^,^^18^ Additionally, the reagents are not standardized, and there are no human anti-AAV calibrators for absolute quantification. Instead, to generate a titer measurement, a dilution series of serum is used to create a dilution-response curve based on the percentage inhibition (or neutralization) with reference to immunodepleted serum (0% transduction) and reporter vector in immunodepleted serum (100% transduction). The calculation of the titer can also differ between assays. The reported titer of NAb assays is usually the dilution of serum that neutralized 50% of vector transduction *in vitro*.^16^ Therefore, an undetectable or negative assay result is not synonymous with an absence of anti-AAV NAbs, and serostatus will consequently be assay dependent. Moreover, the clinical meaningfulness of this surrogate assay and the 50% threshold is not well understood and may vary by AAV serotype. We previously reported successful gene delivery by AAV5 to people with hemophilia B with pre-existing NAbs.^38^ Another study also showed that the presence of AAV5 NAbs did not preclude sustained hepatic expression of factor VIII transgene from the rAAV5 vector valoctocogene roxaparvovec.^15^^,^^39^

Notably, the AAV5 NAb titers prior to gene therapy in this study are higher than a different AAV5 gene therapy study for hemophilia A that excluded participants based on negativity in a total antibody anti-AAV5 binding assay and where patients had either undetectable or low NAb titer prior to dosing.^15^ NAb assay design differences between the studies may contribute to substantially different assay sensitivity (i.e., NAb detection and seropositivity) and titer values; specifically, the MOI used in each assay differs by several fold, and luciferase expression system, luminometer, and sample matrix are not the same. Additionally, despite a similar viral vector, the drug dose and gene payload all contribute to setting the optimal exclusion criteria and thresholds involving AAV antibodies.^1^^,^^2^^,^^38^ Collectively, it is important to use gene therapy product-specific AAV antibody assays for assessing eligibility for a gene therapy product.

Our study has several limitations. The population is small, particularly the subset of participants who were NAb+ at screening but not during the rest of the lead-in period. Consequently, geographical regions and races represented are limited, and this may affect the generalizability of our results to people with hemophilia B globally. We also did not measure antibodies to other AAV serotypes. In addition, although the NAb cell-based TI assay was highly sensitive (LoD: 7), the IgG and IgM ELISAs were comparatively less sensitive (LoD: 50), which limited the comparison between antibodies at lower titers. The study has the advantage of standardized and compliant sample collection and handling procedures, analysis times within verified sample stability, and assays validated for regulated clinical trial use.

This report on the natural history of AAV5 NAbs demonstrated the stability of titer and serostatus, with infrequent seroconversion, over a period of months in the majority of the population screened for the HOPE-B trial and showed that screening for NAbs can occur several months prior to infusion. This insight may be of great utility to clinicians and people with hemophilia B considering gene therapy. Antibody specificity for AAV5 was confirmed by two independent laboratories using different analytical methods and assays validated for regulated clinical trials, and we demonstrated very strong correlation of NAbs with IgG binding antibodies to AAV5. Despite a high correlation, the antibody titer values are not interchangeable between these assays. As our knowledge of the relationship of NAb titer and gene therapy evolves, a sensitive, function-based NAb assay provides unique information. A greater understanding of the natural history of antibodies to gene therapy vectors should be helpful to those considering gene therapy, both clinicians and people with hemophilia B alike.

## Materials and methods

### Study participants

Participants were adult males, aged 18 years or older, diagnosed with inherited hemophilia B classified as either severe (plasma factor IX activity <1 IU/dL) or moderately severe (plasma factor IX activity between 1 and 2 IU/dL) with a severe bleeding phenotype. Key exclusion criteria included a history of factor IX inhibitors, active hepatitis B or C viral infection, and known severe infection or another significant concurrent uncontrolled medical condition. Participants were on stable, continuous prophylaxis with factor IX, with the specific dose and product determined by their physician.

### Study design

HOPE-B was a phase 3, open-label, multinational study that assessed a single dose of etranacogene dezaparvovec in participants with moderate-to-severe hemophilia B. The study was conducted in accordance with International Council for Harmonisation Good Clinical Practice guidelines and ethical principles originating in the Declaration of Helsinki. Prior to gene therapy administration, there was a lead-in period of ≥6 months during which participants received their usual continuous factor IX prophylaxis. During the lead-in phase, visits were planned as follows: one visit occurred approximately 4 weeks after screening (visit L1); subsequent clinic visits were every 2 months until etranacogene dezaparvovec administration criteria were met. The final lead-in visit (L-Final) occurred approximately 4 weeks prior to the planned date of etranacogene dezaparvovec administration. Bleeding/factor use was monitored during the lead-in period. Participants were treated with a single infusion of etranacogene dezaparvovec (2 × 10^13^ genome copies per kg of bodyweight) regardless of pre-existing AAV5 NAbs and will be followed for 5 years. The primary endpoint of HOPE-B was the annualized bleeding rate during a 52-week period from months 7–18 post treatment. Secondary endpoints included factor IX activity levels (measured by the one-stage assay) at 26 and 52 weeks after steady-state factor IX activity was reached as well as factor replacement use, adverse events, and reactive use of corticosteroids.

Pre-treatment serum samples for anti-NAb determination were obtained from participants during the screening period, lead-in period (visit L2 and L-Final), and on the day of etranacogene dezaparvovec administration for the assessment of AAV5 antibodies.

### AAV5 assays

Central laboratories performed the assessments of IgG and IgM AAV5 antibodies (Unilabs, Copenhagen, Denmark) and AAV5 NAbs (Precision for Medicine, Frederick, USA). A cell-based NAb assay with a starting serum dilution of 1:2 and 7 dilution steps was used for both screening and measuring NAb titers. Up to 3 separate assays for each IgG or IgM anti-AAV subclass were used for screening, confirmation, and measuring titers (titering), respectively. The titering assays for IgG or IgM each had a starting serum dilution of 1:50 and 8 dilution steps.

#### AAV5 TI/NAb assay

**Figure 5:**
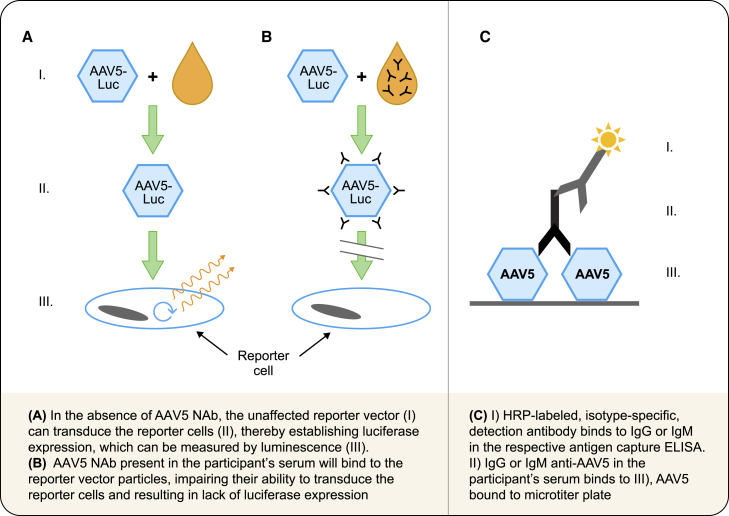
Methods used to measure antibodies to AAV5 AAV5 TI/NAb assay (A and B) and the anti-AAV5 IgG and IgM ELISA (C). AAV5, adeno-associated virus serotype 5; ELISA, enzyme-linked immunosorbent assay; HRP, horseradish peroxidase; IgG, immunoglobulin G; IgM, immunoglobulin M; Luc, luciferase; NAb, neutralizing antibody; TI, transduction inhibition. Reprinted from Liu et al. © 2023 Precision for Medicine.

The TI assay to measure NAbs to AAV5 has been previously described^34^ and is based on an assay principle commonly used in AAV gene therapy (Figures 5A and 5B).^1^^,^^28^ Serial dilutions of heat-inactivated patient serum samples and positive neutralization control (ADK5b mouse monoclonal antibody, Progen, Wayne, PA) samples were prepared to generate a titration curve starting with a 1:2 dilution and proceeding at 3-fold dilution intervals, for a total of seven dilutions, in triplicate wells of a 96-well neutralization plate. Samples with titers greater than the reportable range of the assay starting at a 1:2 dilution were retested starting with a 1:12 starting dilution, followed by six additional 3-fold dilutions. Positive transduction (AAV5(160)-CMV-Luciferase reporter vector in immunodepleted human serum) and negative transduction (immunodepleted human serum and complete media) controls were added to each neutralization plate. The patient samples and positive neutralization control were incubated together with AAV5(160)-CMV-Luciferase reporter vector (uniQure, Netherlands). Aliquots from each well of the neutralization plate were then added to an assay plate with HEK293/T17 reporter cells that had been seeded at 50,000/well and incubated overnight (MOI: 4,200 total particles/cells seeded). Cell viability and transduction were assessed using the ONE-Glo Tox Luciferase Reporter and Cell Viability Assay Kit according to the manufacturer’s instructions (Promega, Maddison, WI), and fluorescence/luminescence was measured using a Biotek Synergy H1 plate reader with GEN5 Software (Agilent, Santa Clara, USA). The percentage neutralization of a sample was calculated from its luminescence signal (i.e., the relative luminescent unit) relative to the average signal of the negative transduction control (representing 0% transduction) and the average signal of the positive transduction control (representing 100% transduction). The test followed a conventional titration approach in which serial dilutions of the test sample were each assessed for neutralizing activity, and the AAV5 NAb titer was the “IC_50_” (midpoint of the titration curve) calculated by 4-parameter regression to percentage neutralization as a function of sample dilution. Testing was conducted at a central laboratory (Precision for Medicine, Frederick, Maryland), and the assay was validated to Clinical Laboratory Improvement Amendments standards with a reportable range of 7–8,748 and intra and inter-run CV ≤30%.

#### Anti-AAV5 IgG and IgM ELISA

The antigen capture ELISAs for IgG and IgM anti-AAV5 measurement were based on previously described methods (Figure 5C).^34^ Briefly, the testing approach comprised tiered screening, confirmatory, and titering assays and was conducted at a central laboratory (Unilabs, Copenhagen, Denmark). For the screening assay, microtiter plates were coated overnight with the drug product (AAV-5-hFDCco-Padua, CSL222, uniQure, Netherlands) and then incubated for 1 h with serum or control samples diluted at 1:50. After a PBS wash, anti-human IgM or IgG conjugated with horseradish peroxidase (HRP) was added. Plates were washed, and tetramethylbenzidine was added to react with HRP, and absorbance was read at 450 nm. A negative control (NC) was created by pooling human serum samples, identified as negative for anti-AAV5 IgG or IgM antibodies, and calculating the percentage inhibition by measuring the samples with and without pre-incubation with etranacogene dezaparvovec. Run-specific cut-off points were calculated based on the pool of NC results and a normalization factor established during validation. Samples with optical density readings above the cut-off point were positive for either IgM or IgG and run in a confirmatory assay.

In the confirmatory assay, 1:50 diluted serum samples were pre-incubated with and without etranacogene dezaparvovec, and the assay was conducted as previously described for the screening assay. The result was expressed as percentage inhibition by etranacogene dezaparvovec in the confirmatory assay compared to the screening assay result and expressed as relative percentage inhibition. The confirmatory cut-off point for a sample positive for IgM anti-AAV5 was ≥45.6% inhibition and for IgG anti-AAV5 was ≥50.4% inhibition, as determined by analyzing 51 samples and the 99% CI.

Positive samples were then run in the titering assay in duplicate. A serum sample was serially diluted in a series of 8 dilutions in buffer (1:50, 1:150, 1:450, 1:1,350, 1:4,050, 1:12,150, 1:36,450, and 1:109,350). The diluted samples were applied to etranacogene dezaparvovec-coated ELISA plates in duplicate wells with the incubation and detection steps as described for the screening assay. A 5-parameter logistic curve fit (Gen 5 software) was used to generate a dilution curve, and the titer was the calculated serum dilution that crossed the assay cut-off point established for the screening assay and adjusted in each run on the basis of the NC and normalization factor (0.082). Testing was conducted at a central laboratory (Unilabs, Copenhagen, Denmark) with a bioanalytical fit-for-purpose assay validation, with intra-run and inter-run precision of ≤30% and measuring range 50–109,350 for both assays.

### Statistics

The analyses detailed in this section describe a retrospective examination of data collected during the lead-in phase of the HOPE-3 (CSL222-061-02) phase 3 pivotal trial and were not specifically powered to detect significant differences or associations. All analyses were performed in SAS 9.4; figures were generated using GraphPad.

Demographic and baseline characteristics were summarized descriptively using sample size (*n*); mean; standard deviation (SD); minimum, maximum, median, and interquartile range for continuous measurements; and frequency and percentages (%) for categorical variables. Continuous baseline covariates were compared between NAb+ and NAb− using two-sample t tests, and the chi-square test was used for categorical associations. Fisher’s exact test was substituted as needed when expected counts were less than 5. The duration of collection time was calculated as the number of days between screening and the last known neutralizing AAV5 titer measurement prior to etranacogene dezaparvovec administration or discontinuation from the lead-in period.

Titer levels were log-transformed when necessary to satisfy normality assumptions in regression analyses and to enhance readability of plots. Coefficient of variation for NAb assay was back-transformed using the correct formula for reporting and interpretation of results.^40^ Assessments from unscheduled visits were excluded from all analyses.

The association between NAb seropositivity and history of hepatitis infection (B or C) was explored using a logistic regression model, controlling for age as a continuous variable, and including an interaction term of age and hepatitis infection. A simple linear regression model was used to evaluate the relationship between NAb titer (dependent variable) and age at screening. NAb titer and IgG were correlated at screening, lead-in month 2 (L2), lead-in month 4 (L4), lead-in month 6 (L6), L-Final, and pre-dose baseline (visit D) using Pearson’s correlation coefficient and 95% CIs. Two anti-AAV5 IgG values from two participants were excluded, both collected 173 days after screening due to suspected laboratory error. The outlying values were 100-fold different (higher or lower) compared to each participant’s other anti-AAV5 IgG values. A variation in anti-AAV5 IgG values of this magnitude was not observed for any other participant and was not considered biologically likely. Intra-assay variability of NAb seropositivity from screening to visit D was assessed with Cohen’s simple kappa and 95% CIs. For participants who maintained seropositivity between screening and visit D, an intraclass correlation coefficient and 95% CIs were calculated using type 3 sums of squares from an analysis of covariance model to assess intra-patient variability of the titer level.

## Data availability

External researchers who would like to obtain individual patient data and any relevant supporting clinical documents for independent research purposes will be asked to complete a Research Proposal Form in order for CSL to evaluate the request. Please note that only anonymized patient data will be provided. The Research Proposal will be evaluated by an internal review committee in order to assess criteria such as the scientific merit of the proposed research, planned analysis, and patients’ informed consent. If a request is declined, a subsequent independent review will be conducted. Data for the study reported herein will remain available for 35 years following regulatory approval. Any data requests should be sent to this email address: office.cmo@cslbehring.com.

## Acknowledgments

This study was funded by CSL Behring. Editorial assistance was provided by Melody Watson of Bioscript Group, Macclesfield, UK, and funded by CSL Behring. Travis Harris from Precision for Medicine contributed to manuscript content.

This work was performed in the USA (Ann Arbor, MI; Aurora, CO; Buffalo, NY; Chapel Hill, NC; Houston, TX; Indianapolis, IN; Little Rock, AK; Los Angeles, CA; Memphis, TN; Nashville, TN; New Haven, CT; Portland, OR; Salt Lake City, UT; San Diego, CA; Seattle, WA; Tampa, FL; and Washington, DC), Belgium (Brussels and Leuven), Denmark (Copenhagen), Germany (Berlin and Frankfurt), Ireland (Dublin), Italy (Florence and Napoli), the Netherlands (Amsterdam, Groningen, Rotterdam, and Utrecht), Sweden (Malmö), and the UK (Cambridge, London, and Southampton).

## Author contributions

R. Klamroth contributed to the study investigation, visualization, and review and editing of the draft; M.R. contributed to the study investigation, visualization, and review and editing of the draft; N.S.K. contributed to the study investigation, visualization, and review and editing of the draft; W.M. contributed to the study investigation, visualization, and review and editing of the draft; S.W.P. contributed to the study investigation, visualization, and review and editing of the draft; R. Kaczmarek contributed to study visualization and review and editing of the draft; D.D. contributed to the study investigation, visualization, writing of the original draft, and review and editing of the draft; B.S. contributed to study visualization and review and editing of the draft; S.L.Q. contributed to study visualization and review and editing of the draft; P.E.M. contributed to study conceptualization, formal analysis, methodology, and review and editing of the draft; N.G. performed formal analyses and contributed to methodology, study visualization, and writing – review and editing of the original draft; P.v.d.V. contributed to the study investigation, visualization, and review and editing of the draft; and J.T. contributed to study conceptualization, formal analyses, and writing of the original draft and review and editing of the draft, methodology, data curation, and project administration.

## Declaration of interests

R. Klamroth has received grant/research support from Bayer, CSL Behring, Novo Nordisk, Octapharma, and Sobi; consultation/speaker fees from Bayer, Biomarin, Biotest, CSL Behring, Chugai, LFB, Novo Nordisk, Kedrion, Octapharma, Pfizer, Roche, Sanofi, Sobi, and Takeda/Shire.

M.R. has received research support from Bayer, BioMarin, CSL Behring, Genentech, Grifols, Hema Biologics, LFB, Novo Nordisk, Octapharma, Sanofi, Spark, Takeda, and uniQure; consultancy fees from Catalyst Biosciences, CSL Behring, Genentech, Hema Biologics, Kedrion, Novo Nordisk, Pfizer, Sanofi, Takeda, and uniQure; and sits on the Board of Directors for the Foundation for Women and Girls with Blood Disorders, and Partners in Bleeding Disorders.

B.S., D.D., S.L.Q., P.E.M., and N.G. are employees of CSL Behring; J.T. was an employee of CSL Behring at the time of research.

R. Kaczmarek has received research funding from Bayer and consulting or lecture fees from Bayer, BioMarin, Spark, Novo Nordisk, and Pfizer.

W.M. has received grant/research support from Bayer, Biotest, CSL Behring, LFB, Novo Nordisk, Octapharma, Pfizer, and Takeda/Shire; consultation/speaker fees from Bayer, BioMarin, Biotest, CSL Behring, Chugai, LFB, Novo Nordisk, Octapharma, Pfizer, Roche, Sobi, and Takeda/Shire; and consultation fees from Bayer, BioMarin, Biotest, CSL Behring, Chugai, Freeline, LFB, Novo Nordisk, Octapharma, Pfizer, Regeneron, Roche, Sanofi, Sobi, Takeda/Shire, and uniQure.

S.W.P. has received consultancy fees from ApcinteX/Centessa, ASC Therapeutics, Bayer, BioMarin, CSL Behring, HEMA Biologics, Freeline, LFB, Novo Nordisk, Pfizer, Poseida Therapeutics, Regeneron/Intellia, Roche/Genentech, Sanofi, Takeda, Spark Therapeutics, and uniQure; research funding from Siemens and YewSavin; and holds a membership on a scientific advisory committee for Equilibra Bioscience and GeneVentiv.

N.S.K. has received grant/research support and consultant fees from uniQure, BioMarin, and Novo Nordisk.

P.v.d.V. has received consultation fees from Bayer.
